# Prevalence of Selected Zoonotic and Vector-Borne Agents in Dogs and Cats on the Pine Ridge Reservation

**DOI:** 10.3390/vetsci4030043

**Published:** 2017-09-04

**Authors:** A. Valeria Scorza, Michael R. Lappin

**Affiliations:** Center for Companion Animal Studies, Department of Clinical Sciences, College of Veterinary Medicine and Biomedical Sciences, Colorado State University, Fort Collins, CO 80523, USA; michael.lappin@colostate.edu

**Keywords:** enteric zoonotic parasites, vector-borne agents, dogs, cats, Pine Ridge Reservation

## Abstract

The prevalence of intestinal parasites and vector-borne agents of dogs and cats in the Pine Ridge Reservation, South Dakota were determined. Fecal samples (84 dogs, 9 cats) were examined by centrifugal floatation and by immunofluorescence assay (FA) for *Giardia* and *Cryptosporidium*. PCR was performed on *Giardia* [beta-giardin (bg), triose phosphate isomerase (tpi), glutamate dehydrogenase genes (gdh)] and *Cryptosporidium* [heat shock protein-70 gene (hsp)] FA positive samples. Cat sera (*n* = 32) were tested for antibodies against *Bartonella* spp., *Toxoplasma gondii*, and FIV, and antigens of FeLV and *Dirofilaria immitis*. Dog sera (*n* = 82) were tested for antibodies against *T. gondii*, *Borrelia burgdorferi*, *Ehrlichia canis*, and *Anaplasma phagocytophilum* and *D. immitis* antigen. Blood samples (92 dogs, 39 cats) were assessed by PCR for amplification of DNA of *Bartonella* spp., *Ehrlichia* spp., *Anaplasma* spp., haemoplasmas, and *Babesia* spp. (dogs only). The most significant results were *Giardia* spp. (32% by FA), *Taenia* spp. (17.8%) and *Cryptosporidium* spp. (7.1%). The *Giardia* isolates typed as the dog-specific assemblages C or D and four *Cryptosporidium* isolates typed as *C. canis*. Antibodies against *T. gondii* were detected in 15% of the dogs. Antibodies against *Bartonella* spp. and against *T. gondii* were detected in 37.5% and 6% of the cats respectively. FeLV antigen was detected in 10% of the cats.

## 1. Introduction

The Pine Ridge Indian Reservation is an Oglala Sioux Native American reservation located in South Dakota. Native American reservations have been recognized as one of the six main distressed regions of poverty in the United States [1]. The prevalence of zoonotic enteric pathogens in client-owned dogs in the United States has been well documented and people are known to be at risk but this information is unknown in the dogs on reservations in the United States [2]. A national survey based on microscopic examination of 1,199,293 fecal samples from client-owned dogs identified the following parasites: ascarids (2.2%), hookworms (2.5%), whipworms (1.2%), *Giardia* (4.0%), and *Cystoisospora* (4.4%) [2]. Additionally, a study done in our laboratory detected *Cryptosporidium parvum* (3.8%) and *Giardia* spp. (5.4%) in client-owned dogs from Colorado [3]. *Giardia* and *Cryptosporidium* are important causes of diarrhea in humans and animals worldwide. Dogs can harbor strains of *Giardia* and *Cryptosporidium* that are dog-specific and also other strains that can be transmitted to humans [4,5]. The prevalence rate of *Cryptosporidium* and *Giardia* in dogs and cats in the United States ranges between 2–10% and 8%, respectively [5,6]. Other parasites commonly harbored by dogs can produce clinical illness in people who may come into contact with dog and cat feces or contaminated environments. *Toxocara canis* can induce visceral larva migrans and *Ancylostoma* spp. can cause cutaneous larva migrans [2]. In the USA, control of these pathogens in domestic animal populations is largely due to prophylactic deworming. However, in economically depressed areas, such as Native American reservations, this practice is not as common. Some prevalence data regarding vector-borne agents in dogs in Native American reservations is available. One survey reported a seroprevalence of 0.1% (1 of 962) and 0.3% (1 of 358) for *D. immitis* and *B. burgdorferi* in dogs of South Dakota [7]. A subsequent study described a similar seroprevalence of *D. immitis* of 1.3% (3 of 234) in dogs from the reservations of South Dakota [8]. However, information regarding vector-borne agents in dogs and cats of the reservation is still limited. The objective of this study was to estimate the prevalence of intestinal parasites and vector-borne agents of dogs and cats in the Pine Ridge Reservation.

## 2. Materials and Methods

Samples from dogs (84 fecal, 82 sera, 92 blood) and cats (9 feces, 32 sera, 39 blood) attending a spay-neutering clinic in Pine Ridge Reservation, South Dakota were included in the study. Pine Ridge Reservation has a steppe climate with cold winters and warm summers. The annual high temperature is 62.1°F and the annual low temperature is 32.1°F. Steppes are semi-arid (the average annual precipitation is 18 inches) and most of the precipitation falls during the summer [9].

Animals were entered into the study regardless of their health status. Use of anthelmintic, *D. immitis* preventatives, and vector control was unknown but believed by the organizers to be unlikely.

Fecal samples were examined for parasites by microscopic examination after Sheather’s sugar centrifugation and for *Giardia* and *Cryptosporidium* by a commercial immunofluorescence assay (Merifluor *Crypto/Giardia* kit, Meridian Diagnostic Corporation, Cincinnati, OH, USA). Prior to immunofluorescence assay (FA) and DNA extraction, all fecal samples were concentrated using sugar concentrating techniques as previously described [10]. Total DNA was extracted from the *Cryptosporidium* spp. and *G. duodenalis* FA positive samples as described [11]. PCR amplification was performed in the FA positive samples as published using the beta-giardin (bg), triose phosphate isomerase (tpi), and glutamate dehydrogenase (gdh) genes for *Giardia* and the heat shock protein-70 (hsp) gene for *Cryptosporidium* [12,13,14,15,16]. The DNA sequences were examined in forward and reverse direction with an ABI3100 Genetic Analyzer (Applied Biosystems, Foster City, CA, USA). The sequence data were compared with those in the Genbank^®^ database by BLAST analysis (http://blast.ncbi.nlm.nih.gov/) to determine the *Cryptosporidium* and *Giardia* species.

Serum from dogs was assayed by a commercially available ELISA (SNAP^®^ 4Dx test; IDEXX Laboratories, Westbrook, ME, USA) for simultaneous qualitative detection of *Dirofilaria immitis* antigen, and antibodies against *Borrelia burgdorferi, Ehrlichia canis*, and *Anaplasma phagocytophilum.* Additionally, dog serum was evaluated for *Toxoplasma gondii*-specific antibodies by ELISA (Veterinary Diagnostic Laboratory, Colorado State University, Fort Collins, CO, USA). Serum from the cats was tested for specific antibodies by ELISA (*Bartonella henselae*, and *Toxoplasma gondii*) [17,18]. Cats were screened for *D. immitis* antigen, feline immunodeficiency virus antibody, and feline leukemia virus antigen by a commercially available ELISA (SNAP Feline Triple Test, IDEXX Laboratories, Westbrook, ME, USA). Total DNA was extracted from blood samples of cats and dogs for PCR assays for *Rickettsia* spp., *Bartonella* spp., *Ehrlichia/Anaplasma*/and *Hemoplasma* spp. [19,20,21,22]. Additionally, dogs were also screened for the presence of *Babesia canis* DNA by PCR [23]. Overall prevalence for parasite infections in dogs and cats was defined as the percentage of fecal samples that tested positive for any parasite by any of the diagnostic tests. Specific parasite prevalence rates were also included.

## 3. Results

Prevalence for internal parasites is displayed in Table 1. Sixteen of 84 dogs (19%) had dual infections and six had triple infections (7.1%). No enteric parasites were detected in cats.

Four of the six *Cryptosporidium* spp. FA positive isolates typed as *Cryptosporidium canis.*

Twenty-seven samples were positive for *Giardia* spp. by FA: nine (33.3%), eight (29.6%), and three (11.1%) samples were amplified by the bg, gdh and tpi genes respectively (Table 2). The positive amplicons have been deposited in the Genbank^®^. The *G. duodenalis* accession numbers are included in a larger study that genotyped *G. duodenalis* isolates from dogs from the United Stated [24].

Antibodies against *T. gondii* were detected in 12 of the 82 (14.6%) dog sera but all sera were negative for other antibodies as well as *D. immitis* antigen. Three of the twelve samples that tested positive for *T. gondii* tested positive for gastrointestinal parasites including *G. duodenalis* and *Taenia*. Antibodies against *Bartonella henselae* and *T. gondii* were detected in 12 of 32 (37.5%) and in 2 of 32 (6%) of the cat sera respectively. FeLV antigen was detected in four of 39 (10.2%) of the cats but all were negative for *D. immitis* antigen and FIV antibodies. None of the 92 dog blood samples and 39 cat blood samples was PCR positive for DNA of the target agents. Two of the 12 samples that tested positive for *Bartonella* antibodies tested also positive for FeLV antigen.

## 4. Discussion

Overall enteric parasites in dogs and *Bartonella* spp. antibodies in cats were common on the Pine Ridge Reservation. These results are expected since most of these animals are free roaming and do not have regular access to veterinary care and most of them consume raw meat diets. The prevalence of *Giardia* spp. among the dogs in the reservation is higher than the average in the U.S. Prevalence estimates in domestic dogs in the U.S. range anywhere from 4% to 29% depending on the type of test used and on the study population [2,25]. The high prevalence detected by FA (32%) in comparison to fecal flotation (11.9%) is due to the higher sensitivity and specificity of the FA assay for *Giardia* and *Cryptosporidium* in dogs [26]. All the *Giardia* isolates that were successfully genotyped were dog-specific assemblages C or D. This finding is consistent with previous reports; a review article described that 67% (1049 of 1563) isolates from dogs worldwide were either Assemblage C or D [27]. Other studies also reported that Assemblages C and D mostly prevail in dogs from Cambodia, Croatia, Germany, Italy and the United States [24,25,28,29,30,31]. However, a study from the western U.S. in reported that multiple infections with zoonotic assemblages were most commonly detected [32]. It was also expected that most of the dogs harbor dog-specific assemblages since intensive contact between large numbers of dogs favours the transmission of dog-specific isolates [25,31,33,34]. Different amplification rates among the genes are expected since the PCR assays used in the study do not have the same analytical sensitivity and specificity [24,28,31,32]. The prevalence of *Cryptosporidium* in dogs in the United States could be as high as 17% so the prevalence detected in this study was under the expected range [5]. Dogs are usually infected with *C. canis* and humans are usually infected with *C. hominis* and *C. parvum senso stricto*. Occasionally, *C. canis* have been also detected in human feces; of a total of 22,505 samples from humans from 40 countries, only 4 were *C. canis* (0.02%) [4,35].

The *Taenidae* spp. infection in the dogs was high and expected since the diet of dogs in the reservation is based on raw meat. *Toxoplasma gondii* is also commonly transmitted in the tissues of intermediate hosts. Approximately 30% of cats and dogs in the United States have *T. gondii* antibodies [36]. Rural and stray dogs can act as sentinels of *T. gondii* because they are carnivores and they can mechanically transmit oocysts to humans after ingesting sporulated oocysts in feline feces [37]. The seroprevalence in dogs worldwide ranges anywhere from 5% up to 45.3% [37,38,39,40].

We believe that no enteric parasites were detected in cat fecal samples due to the very small number of samples tested in the study. However, results might also be false negatives due to shedding of parasites below the detection limit of the test.

The seroprevalence of *T. gondii* in cats and dogs on the reservation was 6% and 15% respectively, which was lower than expected considering that these animals are fed a raw meat diet. Thus, cats and dogs in this reservation do not appear to be more likely to shed or mechanically transmit *T. gondii* into the human environment than pet dogs in other environments. It was demonstrated that dogs can transmit *T. gondii* oocysts after eating cat feces, but dogs skin temperatures are not suitable for non-sporulated oocysts to sporulate [41]. However, the effect of sporulated oocyst in the skin of dogs has not been studied [42]. Previous studies reported that cystic echinococcosis has been endemic among the Navajo, Zuni and Santo Domingo Indians due to an enzootic dog–sheep cycle on the reservations [43,44,45]. However, *Echinococcus* spp. eggs cannot be differentiated morphologically from those of *Taenia* spp. [46]. Therefore, it may be possible that some of the *Taenia* spp. identified could be *Echinococcus* spp. eggs.

The average seroprevalence of *Bartonella henselae* in cats in the United States and in the Rocky Mountain-Great Plains regions are 27.9% and 4%, respectively [47]. However, cats of the Pine Ridge reservation were not included in the previous study [47]. In the current study, 37.5% of the cats had antibodies reacting to *B. henselae* and all of the cats were negative for *Bartonella* spp. by PCR, suggesting the bacteremia had been limited by the time of sample collection. This finding is similar to the one seen in cats in California where 43.5% have antibodies to *B. henselae* but were not bacteremic [48]. Exposure to *B. henselae* is most prevalent in temperate regions; Pine Ridge reservation has a humid continental climate with hot summers and no dry season that can favor the presence of *Ctenocephalides felis*, the vector of *B. henselae*. While data on *C. felis* infestation rates were not available, these data suggest infestation was common and that flea control is indicated if possible.

In contrast, all the dogs were negative for the select vector-borne agents. The most important tick-borne rickettsial diseases of dogs and people in the United States are anaplasmosis, ehrlichiosis and Rocky Mountain Spotted Fever. The tick vectors responsible for the transmission of these diseases are *Amblyomma americanum*, *Dermacentor variabilis*, *Ixodes* spp. and *Rhiphicephalus sanguineus*. The most common tick vector present in South Dakota is *R. sanguineus,* which can be a vector for *Babesia vogeli*, *E. canis*, and *Rickettsia rickettsii*. A larger serological survey in the USA reported that none of the 358 dogs tested in South Dakota had antibody titers against *Ehrlichia* spp. (*E. canis* and *E. chaffeensis*) but no information regarding anaplasmosis was reported [48]. Unfortunately we were not able to collect ticks from the dogs of the study to confirm the presence of these pathogens. In addition, we only screened for *Rickettsia* spp. and *B. vogeli* infections by PCR assay which is generally only positive during the acute stages of infection and so prevalence rates for these agents could be underestimated.

The *D. immitis* negative results observed in this study correlate with previous findings; two studies reported prevalence rates of 0.013% and 0.1% [8,49]. FeLV and FIV are retroviruses that cause immunosuppression in cats; prevalence ranged from 2% to 18% [50]. In this study, FeLV was detected in 10% of the cats and FIV was not detected. The prevalence of FIV is low in indoor cats or in rural regions where the cat population density is low. Therefore, cats in the reservation do not appear to be at a high risk of infection.

## 5. Conclusions

Although the sample set was relatively small, enteric parasites in dogs and *Bartonella* spp. antibodies in cats were common. To our knowledge, this is the first study that assesses the prevalence of enteric zoonotic parasites and vector-borne agents in dogs and cats from the Pine Ridge Reservation. This information should be used to develop a preventive medicine plan that could be implemented for the dogs and cats on this, and similar reservations.

## Figures and Tables

**Table 1:** Prevalence of gastrointestinal parasites in dogs (*n* = 84) of Pine Ridge Reservation, South Dakota by fecal flotation and by immunofluorescence assay (FA) for *Giardia* spp. and *Cryptosporidium* spp.

Parasites	No of Positives	Prevalence (%)
Overall	40	47.6
*Taenidae* spp.	15	17.9
*Giardia* spp.	10 **^a^**	11.9 **^a^**
	27 **^b^**	32.1 **^b^**
*Cryptosporidium* spp.	6 **^b^**	7.1 **^b^**
*Toxascaris leonina*	8	9.5
*Toxocara canis*	6	7.1
Hookworms	3	3.6
*Cystoisospora canis*	2	2.3

**^a^** Fecal flotation microscopic examination after Sheather’s sugar centrifugation; **^b^** Merifluor *Crypto/Giardia* kit, Meridian Diagnostic Corporation, Cincinnati, OH, USA.

**Table 2:** *Giardia duodenalis* assemblages in dogs in Pine Ridge Reservation, South Dakota by GDH, TPI, and BG genes.

# of Genes Amplified	# Dogs	GDH	TPI ^b^	BG
1 (11) **^a^**	1	-	-	D
	3	-	-	C
	2	C	-	-
	2	D	-	-
2 (2) **^a^**	2	D	-	C
	1	C	D	-
	1	-	D	C
	1	-	D	D
	1	C	-	D

**^a^** Number of animals that tested positive for that number of genes tested; **^b^** includes TPI-generic and dog-specific primers.
